# 5,6,7,4′‐Tetramethoxyflavone, a Dietary Polymethoxyflavone, Exerts Antitumor Effect on HeLa Xenografts in Mice

**DOI:** 10.1002/fsn3.71158

**Published:** 2025-10-31

**Authors:** Qiang You, Haiyan Ding, Dan Li, Yuan Hu, Youping Liu

**Affiliations:** ^1^ Department of Pharmacy Hospital of Chengdu University of Traditional Chinese Medicine Chengdu China; ^2^ Department of Pharmacy The Second Affiliated Hospital of Hainan Medical University Haikou China; ^3^ School of Pharmacy Chengdu University of Traditional Chinese Medicine Chengdu China

**Keywords:** 5,6,7,4′‐tetramethoxyflavone, cervical cancer, network pharmacology, polymethoxyflavone, proteome microarray, transcriptomics

## Abstract

5,6,7,4′‐Tetramethoxyflavone (TMF), a naturally occurring polymethoxyflavone (PMF) abundant in *Citrus* species, has demonstrated potent antitumor activity against HeLa cells in vitro. To extend these findings, this study systematically investigated its therapeutic efficacy and safety profile in a HeLa tumor xenograft model. Here, we first found that TMF induced apoptosis in HeLa cancer cells both in vitro and in vivo. Proteomics analysis identified 19 differentially expressed proteins (DEPs) from HeLa cancer cells after TMF treatment, including downregulation of HSP60, sTNF‐R1, JNK, TAK1 (S412), TBK1 (S172), ZAP70 (Y292), ATF2, c‐Fos, c‐JUN, Smad1, Smad5, and Stat6 (Tyr64), alongside upregulation of sTNF‐R2, AKT, GSK3b, MKK3, MKK6, MSK2, and P38. Transcriptomics analysis further uncovered that there were 261 differentially expressed genes (DEGs). Kyoto Encyclopedia of Genes and Genomes (KEGG) enrichment for the 19 DEPs and 261 DEGs revealed that the underlying mechanisms of TMF against HeLa tumor pointed primarily to MAPK, TNF, VEGF, Ras, and FoxO signaling pathways. Notably, histopathological evaluation revealed no observable tissue damage in major organs (liver, kidney, lung, heart, spleen) following TMF administration. In addition, the results of biochemical indexes further verified that the overall changes in plasma ALT, AST, TBIL, DBIL, TRIG, ALP, LDH, GGT, CREA, UA, UREA, CK, HBD, and CHOL were significantly smaller in the TMF‐treated groups than in the DDP‐treated groups. These findings collectively position TMF as a promising therapeutic candidate combining robust antitumor efficacy with favorable safety characteristics for the treatment of cervical cancer.

## Graphical abstract

5,6,7,4′‐Tetramethoxyflavone can effectively inhibit the growth of cervical cancer transplanted tumors in nude mice, and it exhibits less toxicity, superior to cisplatin in safety profile.

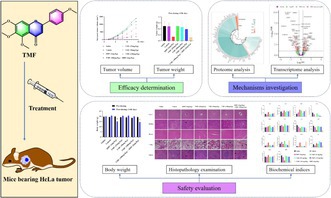

**Abbreviations**
ALP: alkaline phosphatase
ALT: alanine aminotransferase
AST: aspartate aminotransferase
CCA: cervical cancer
CHOL: cholesterol
CK: creatine kinase
CREA: creatinine
DBIL: direct bilirubin
DDP: cis‐platinum
DEGs: differentially expressed genes
DMSO: dimethyl sulfoxide
GGT: γ‐glutamyl transpeptidase
GO: Gene Ontology
HBD: hydroxybutyrate dehydrogenase
KEGG: Kyoto Encyclopedia of Genes and Genomes
LDH: lactate dehydrogenase
PMF: polymethoxyflavone
PPI: protein–protein interaction
TBIL: total bilirubin
TMF: 5,6,7,4′‐tetramethoxyflavone
TRIG: triglyceride
UA: uric acid

## 1 Introduction

Cervical cancer (CCA) remains the most prevalent gynecological malignancy worldwide, with an estimated annual global burden of 662,301 new cases and 348,874 related deaths (World Health Organization 2020). Despite significant advancements in preventive strategies including HPV vaccination programs and improved screening techniques, coupled with evolving therapeutic approaches, CCA continues to demonstrate rising incidence and mortality rates (Arbyn et al. 2020). Current clinical management relies heavily on chemotherapeutic agents such as cisplatin, paclitaxel, and 5‐fluorouracil, which serve as the cornerstone of comprehensive treatment regimens, particularly for advanced‐stage and metastatic disease. However, the therapeutic efficacy of these first‐line agents is substantially compromised by dose‐limiting toxicities (including myelosuppression, hepatorenal impairment, severe gastrointestinal complications, cardiotoxicity, and peripheral neuropathy) (Yu et al. 2017). These clinical challenges contribute to suboptimal treatment outcomes, reflected in the dismal 5‐year survival rates below 30% for advanced CCA patients (Liu 2018). Therefore, this critical situation underscores the urgent need for developing novel chemotherapeutic agents with enhanced therapeutic indices and reduced systemic toxicity profiles and the emergence of chemoresistance.

**FIGURE 1:**
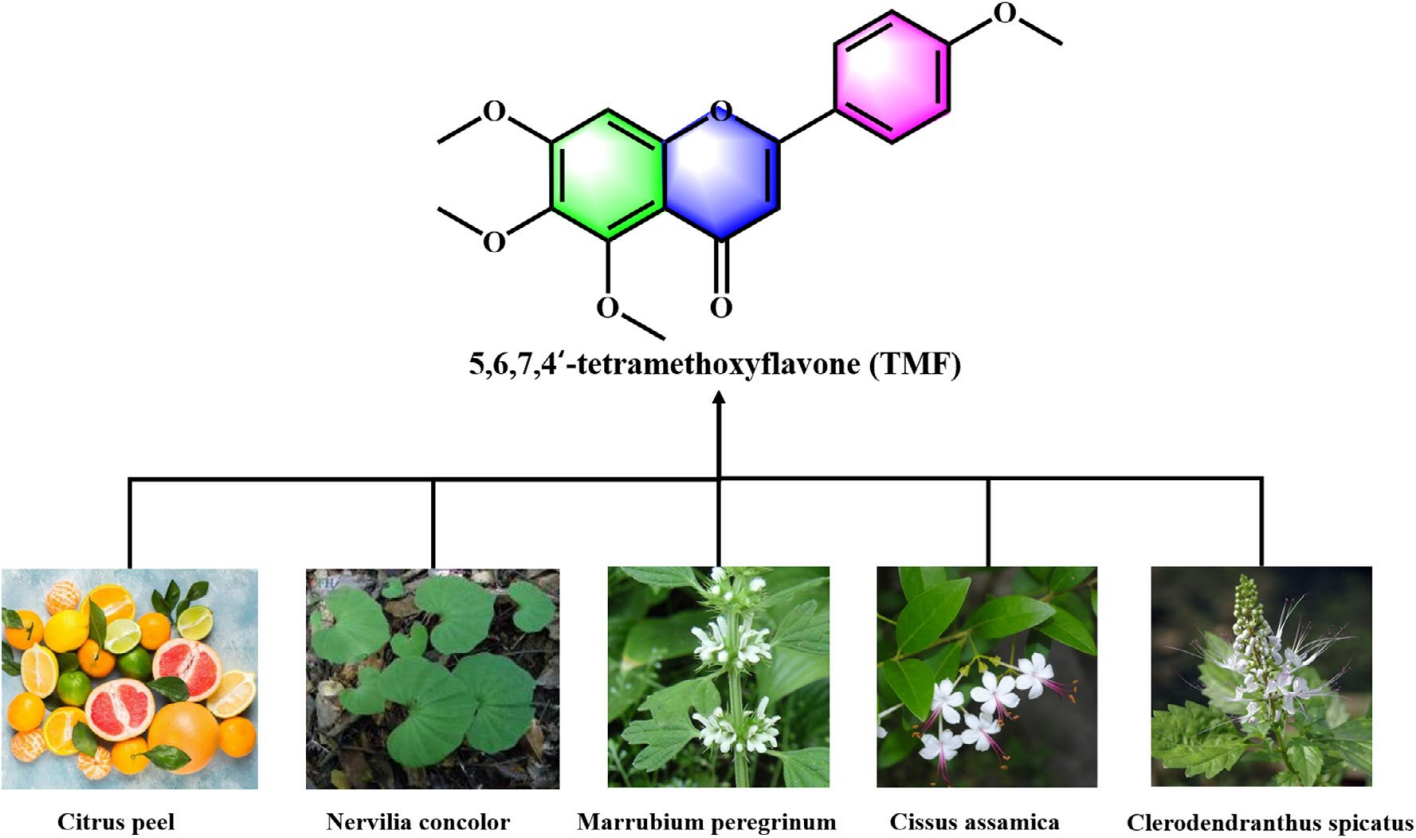
Chemical structure of 5,6,7,4′‐tetramethoxyflavone (TMF) and its natural sources including *Citrus* peel, *Nervilia concolor*, *Marrubium pergrinum*, *Cissus assamica*, and *Clerodendranthus spicatus*.

5,6,7,4′‐Tetramethoxyflavone (TMF) is a naturally occurring polymethoxyflavone (PMF) found in multiple genera including *Citrus* germplasms (Peng et al. 2021), *Nervilia concolor* (Tran et al. 2021), *Marrubium pergrinum* (Alkhatib et al. 2010), *Cissus assamica* (Chan et al. 2018) and *Clerodendranthus spicatus* (Chen et al. 2020) (Figure 1). TMF is particularly abundant in various *Citrus* flavedo and has the highest content in *Citrus reticulata* Blanco (7.45 mg/g) that has also frequently been used in traditional Chinese medicine prescriptions for the treatment of indigestion, abdominal fullness and distention, cough, inflammation, and cancer (Yu et al. 2018). Several in vitro studies have reported the potential antitumor activity of TMF. An earlier study showed that TMF exhibited potent cytotoxicity to NCI‐60 cancer cells through inhibition of mitosis mediated by interfering with tubulin polymerization like the mechanism of antitumor action of paclitaxel and vincristine, with a GI_50_ of 28 μM (Beutler et al. 1998). Another study (Manthey and Guthrie 2002) suggested that TMF significantly inhibited the proliferation of DMS‐114, HT‐29, MCF‐7, MDA‐MB‐435, and DU‐145 cancer cells. A subsequent study (Ohtani et al. 2007) found that TMF enhanced the chemosensitivity to vincristine in P‐gp‐overexpressing K562/ADM cancer cells by increasing vincristine uptake via inhibition of P‐gp. In addition, TMF improved the antiproliferative effect of 5‐fluorouracil in HT29 cancer cells by upregulating CDH1 and downregulating ZEB1 and SNAI1 (Pereira et al. 2019). Our recent study (You et al. 2021) revealed that TMF exhibited significant inhibitory activity against HeLa cancer cells, with the maximum blood concentration of 224.52 ± 36.62 ng/mL and AUC of 494.45 ± 79.13 h ng/mL in rats after oral administration.

Taken together, TMF has a potent inhibitory effect on various cancer cells in vitro, where its potential antitumor activity has not been investigated in vivo. The aim of this study was to (1) evaluate the in vivo anti‐HeLa cancer efficacy of TMF using a subcutaneous xenograft model in nude mice, (2) elucidate the mechanism of action of TMF against HeLa tumors through proteomics and transcriptomics, and (3) assess the safety of TMF using histopathology and plasma biochemical indexes.

## 2 Materials and Methods

### 2.1 Chemicals and Reagents

TMF (HPLC > 98%) used for this study was synthesized in our previously published article (You et al. 2021). Cis‐platinum was purchased from Energy Chemical (Shanghai, China). Dimethyl sulfoxide (DMSO) (biochemical grade, BCCD7238) was purchased from Sigma‐Aldrich (Shanghai) Trading Co. LTD. Fetal bovine (20200408) and Trypsin–EDTA (70070200) solution were obtained from Biosharp (Beijing, China). Penicillin–streptomycin (J190033) solution was purchased from HyClone laboratories (Utah, USA). PEG400 (injection grade, 20191009) was purchased from Weier pharmaceutical Co. Ltd (Nanjing, China). Annexin V‐FITC/PI Apoptosis Kit was purchased from Kaiji Biotechnology Co. Ltd (Nanjing, China). Biochemical kits including ALT (46893301), AST (46004201), GGT (49922501), ALP (47881101), TBIL (45664301), DBIL (46042501), CREA (47624401), UA (47878601), LDH (49771201), CK (49845401), UREA (48503001), HBD (47790201), TRIG (47714101), and CHOL (51832701) kit were purchased from Roche Diagnostics (Basel, Switzerland).

### 2.2 Cells Culture

HeLa cells were provided by Chengdu University of TCM. The cells were cultured in DMEM media (Gibco, 8120501) supplemented with 10% sterile fetal bovine serum, 100 U/mL penicillin and 0.1 mg/mL streptomycin solution at 37°C with 5% CO_2_.

### 2.3 Animals

The animal experiment was approved by the Institutional Animal Care and Use Committee of the Chengdu University of TCM (ethical approval number: 2020‐38). Sixty female BALB/c‐nude mice (6 weeks old, weighing 20 ± 2 g, license No: SCXK [Beijing] 2019‐0010) were purchased from SPF Biotechnology Co. Ltd. (Beijing, China). The mice were housed in a specific pathogen‐free animal room (25°C ± 2°C), with relative humidity (40%–70%) and an alternating 12‐h light/dark cycle, with free access to water and high‐protein feed sterilized by Co60 ray for 1 week of acclimation.

### 2.4 HeLa Xenograft Model and Experimental Protocol

The in vivo antitumor activity of TMF on HeLa cancer was evaluated using a HeLa xenograft model, which was established by implanting HeLa cells subcutaneously at a density of 5 × 10^6^ cells/0.2 mL to the right armpit of mice. After the tumors reached 40–70 mm^3^, these 60 mice were randomly divided into seven groups: Saline (*n* = 8), Vehicle (*n* = 8, control, 13.5% DMSO + 36.5% saline + 50% PEG400), DDP (*n* = 8, positive control, 2 mg/kg), TMF (25, 50, and 100 mg/kg) (*n* = 9), and TMF 100 mg/kg + DDP (*n* = 9) groups. All mice were administered intraperitoneally for 15 days. Body weights and tumor volumes of nude mice were measured every other day. Tumor volumes were measured with an electronic vernier caliper and were calculated using the formula *V* (mm^3^) = *ab* ^2^/2, where *a* is the longest superficial diameter and *b* is the smallest superficial diameter.

At the end of the experiment, blood was sampled from the mice's eyes, centrifuged at 3000*g* and 4°C for 10 min, and then the supernatant was transferred to Eppendorf tubes and stored at −20°C. Following blood sampling, the mice were sacrificed, and the tumors, livers, hearts, spleens, lungs and kidneys were necropsied. These tissues were preserved in two ways: in a −80°C refrigerator and in 4% paraformaldehyde solution.

### 2.5 Apoptosis Analysis

The effect of TMF on HeLa cells was determined using an Annexin V‐FITC/PI Apoptosis Kit. Briefly, HeLa cells were seeded in a 12‐well plate at a density of 10,0000 cells/well for 24 h and were treated with TMF at concentrations of 0, 10, 20, and 40 μM/L for another 24 h. The media were removed, and the plate was washed with PBS twice. Then, the cells were collected and treated following the instructions of the apoptosis kit, and the results were analyzed using a flow cytometer (Becton‐Dickinson, FACSVerse, Bedford, USA).

### 2.6 Transcriptome Analysis

The HeLa tumor tissues of the Vehicle group were used as control, while those of the TMF (100 mg/kg) group were used as the experiment. A TRIZOL solution was used to extract RNA, which was quantified by NanoDrop 2000 (Thermo Fisher Scientific, Wilmington, DE). An Agilent Bioanalyzer 2100 system (Agilent Technologies, CA, USA) was used to assess RNA integrity. Transcriptome libraries were constructed using the VAHTS mRNA‐seq V3 Library Prep Kit for Illumina, and RNA was sequenced using the Illumina NovaSeq Platform. After the routine procedures of RNA extraction, library construction for transcriptome sequencing, RNA‐seq sequencing and quality control, differentially expressed genes (DEGs) were screened according to *p* < 0.05, FC ≥ 1.5. Based on the DEGs, protein–protein interaction (PPI) analysis was performed using the Search Tool for the Retrieval of Interacting Genes/Proteins (STRING) online database version 11.5 (https://cn.string‐db.org/) (Szklarczyk et al. 2021), and the network was visualized with Cytoscape version 3.7.1 (www.cytoscape.org/) (Shannon et al. 2003). GO (Gene Ontology) functional annotation and KEGG (Kyoto Encyclopedia of Genes and Genomes) pathway enrichment analysis were performed through DAVID Bioinformatics Resources (https://david.ncifcrf.gov/). An online mapping platform, BioInformaticas (http://www.bioinformatics.com.cn/), was utilized to visualize GO and KEGG results.

### 2.7 Proteome Microarray Analysis

Human Phosphorylation Pathway Profiling Array C55 and Human Apoptosis Array C1 (Raybiotech Inc.) were used to determine the effect of TMF on the HeLa proteome. Preparation of HeLa tumor samples referred to Section 2.6. The proteome was determined in accordance with the manufacturer's instructions. Briefly, the main procedure included protein extraction with Cell Lysis Buffer, determination of protein concentration with BCA Protein Quantitation Kit, blocking and incubation of the antibody membrane, and detection of signals with ImageQuant LAS4000 Scanner (General Electric Company, USA). The detailed operation was referred to the procedure described by Zhang et al. (2020). Differentially expressed proteins (DEPs) were identified through the formula Ln(FC) = |*E* − *C*|, where *E* and *C* represent the average expression level of the experimental group and control group, respectively. FC ≥ 1.2 indicates a marked difference between the two groups. Then, PPI, GO functional annotation and KEGG pathway enrichment analysis were conducted for the DEPs.

### 2.8 Histopathological Analysis

The tissues of three mice of each group were randomly selected for histopathological analysis. After a routine histopathological procedure, including paraformaldehyde fixation, paraffin embedding, sectioning and hematoxylin‐eosin (H&E) staining, histological alterations such as necrosis, apoptosis, swelling, and atrophy were evaluated based on scoring systems previously reported (Mai et al. 2019).

### 2.9 Plasma Biochemical Analysis

The plasma collected from 'Materials and Methods' section 2.4 was used for biochemical analysis. Fourteen biochemical indexes, including *ALT*, *AST*, *TBIL*, *DBIL*, *TRIG*, *ALP*, *LDH*, *GGT*, *CREA*, *UA*, *UREA*, *CK*, *HBD*, and *CHOL*, were determined to evaluate the effect of TMF on liver, kidney and heart function using a fully automatic biochemical analyzer (Cobas c 311, Roche Diagnostics, Basel, Switzerland).

### 2.10 Statistical Analysis

All experiments were repeated three times at a minimum, and the results were presented as mean ± SD. Statistical significance was assessed with a one‐way *ANOVA* test or Student's *t*‐test using SPSS 20. A *p*‐value < 0.05 was considered statistically significant.

## 3 Results

### 3.1 Effect of TMF on Apoptosis in HeLa Cells

**FIGURE 2:**
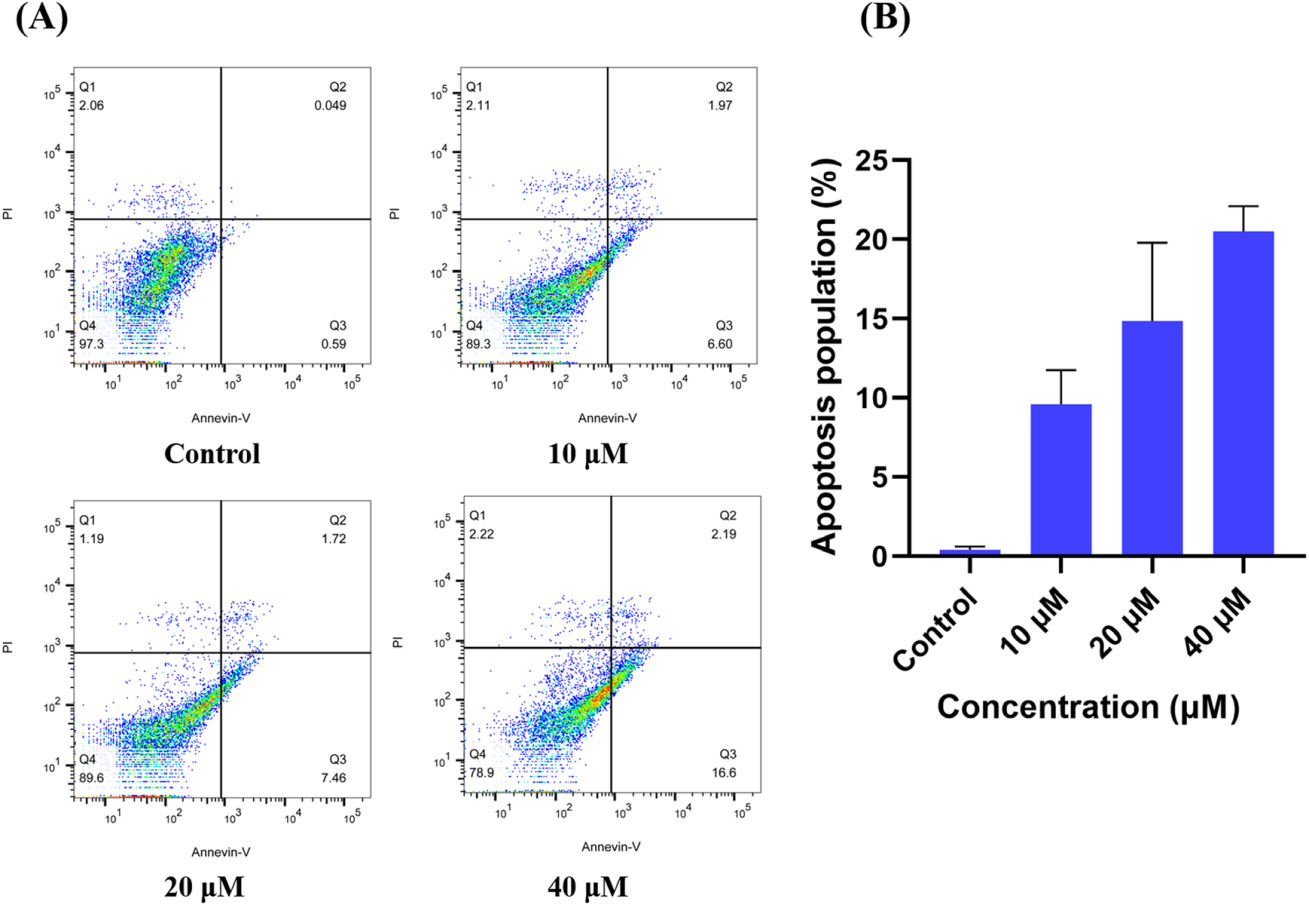
TMF induces early and late apoptosis in HeLa cancer cells. (A) HeLa cells were treated with TMF at concentrations of 0, 10, 20, and 40 μM for 24 h. Flow‐cytometry results indicated that TMF significantly increases Annexin V‐positive and Annexin V/PI‐positive cell populations in a dose‐dependent manner. (B) Graphical representation of apoptosis rates performed in triplicate and expressed as the mean ± standard deviation.

To investigate the apoptotic effect of TMF on HeLa cells, flow cytometry in conjunction with Annexin V‐FITC and PI staining was used. As shown in Figure 2, the apoptotic rates of HeLa cells treated with TMF at concentrations of 0, 10, 20, 40 μM were 0.40 ± 0.17, 9.61 ± 1.76, 14.86 ± 4.03, and 20.54 ± 1.28, respectively. Compared with the control (0 μM), TMF at concentrations of 10, 20, and 40 μM significantly increased the apoptotic rates (*p* < 0.05), and the percentage of apoptotic cells increased with increasing concentrations of TMF, indicating a dose–response relationship.

### 3.2 Effect of TMF on Tumor Cell Growth in Nude Mice Bearing HeLa Xenograft

To evaluate the tumor‐inhibitory effect of TMF in vivo, a subcutaneous HeLa xenograft model was established. Between the first dose and the last dose, 5 mice died, and finally 55 mice completed the experiment, including Saline (*n* = 8), Vehicle (*n* = 8), DDP (2 mg/kg, *n* = 6), TMF (25 mg/kg) (*n* = 9), TMF (50 mg/kg) (*n* = 9), TMF (100 mg/kg) (*n* = 9), and TMF (100 mg/kg) + DDP (2 mg/kg) (*n* = 6) group.

**FIGURE 3:**
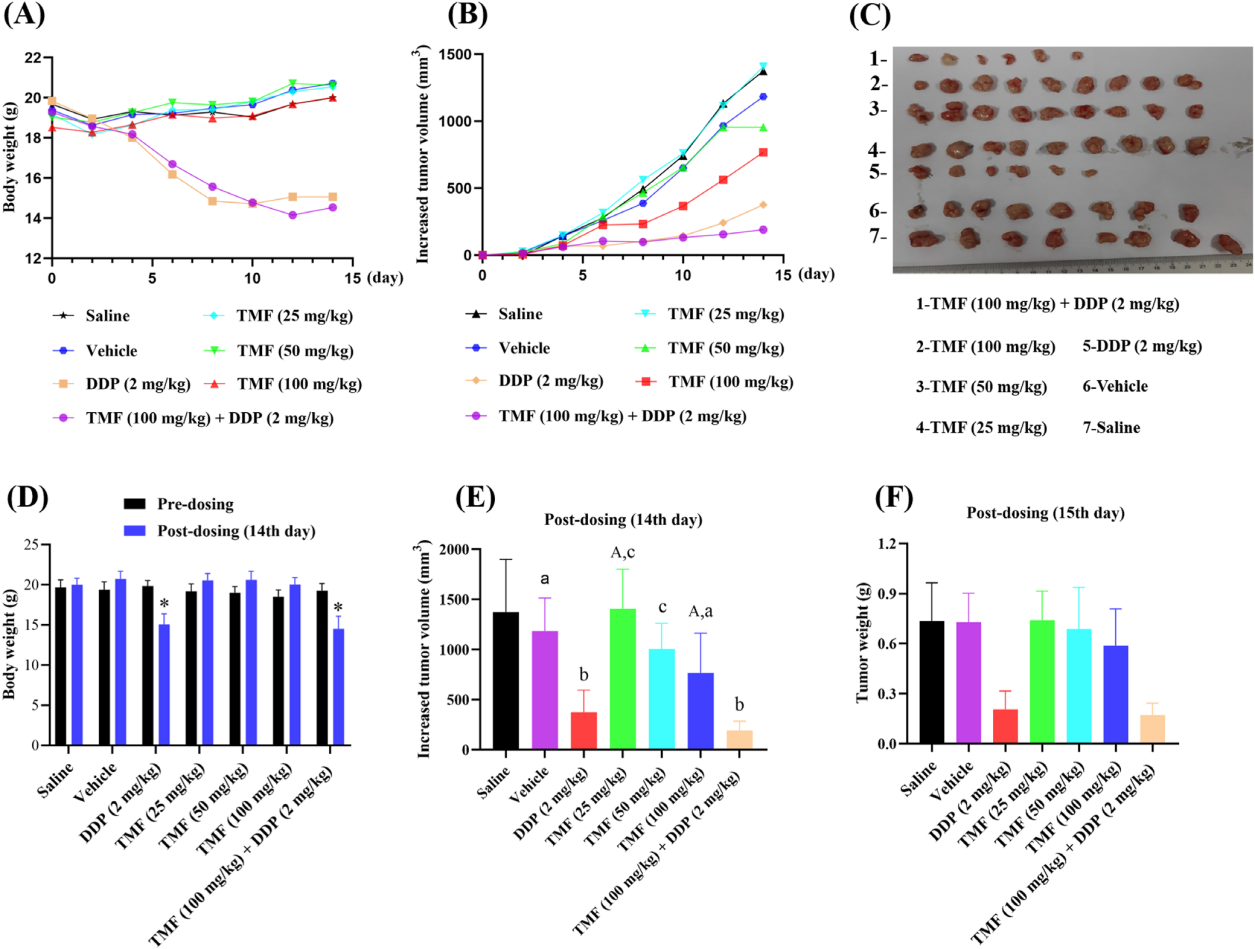
Antitumor effect of TMF on cell growth in nude mice bearing HeLa xenograft. (A) Line chart of body weight of mice and (D) Comparison of body weight of mice between pre‐dosing and post‐dosing (14th day); (B) Line chart of increased tumor volume of mice and (E) Bar graph of increased tumor volume on the 14th day; (C) Tumors removed from nude mice and (F) Bar graph of tumor weight.

As shown in Figure 3A,D, the mean body weight of mice in the DDP (2 mg/kg) group and TMF (100 mg/kg) + DDP (2 mg/kg) group showed a downward trend gradually over time, while the other groups showed an upward trend. The mean body weight had a statistically significant difference between vehicle‐treated mice and DDP (2 mg/kg)‐treated mice (*p* = 0.000), whereas it did not differ significantly between vehicle‐treated mice and the TMF (25 mg/kg) group or the TMF (50 mg/kg) group or the TMF (100 mg/kg) group (*p* > 0.05). In addition, the body weight of mice in the vehicle group did not change significantly compared with the saline group. The above results indicated that DDP inhibited the growth of nude mice, while vehicle and TMF did not inhibit.

By comparing the tumor volume before administration, the increased tumor volume was measured after administration to reflect the efficacy. As shown in Figure 3B,E, the increased tumor volume of the seven groups gradually increased over time, while the comparison of increase rate among the seven groups was approximately saline group ≈ vehicle group ≈ TMF (25 mg/kg) group > TMF (50 mg/kg) > TMF (100 mg/kg) > DDP (2 mg/kg) > TMF (100 mg/kg) + DDP (2 mg/kg) group. After the last dose, there was no significant difference between saline group and vehicle group (*p* = 0.4), suggesting that vehicle had no effect on the tumor growth. Compared with the control (vehicle), a dose of TMF (25 mg/kg) (*p* = 0.24) and TMF (50 mg/kg) (*p* = 0.35) did not significantly suppress tumor growth, while TMF (100 mg/kg) resulted in a significant reduction of tumor growth (*p* = 0.03). No significant difference was observed between the DDP (2 mg/kg) group and TMF (100 mg/kg) + DDP (2 mg/kg) group. The increased tumor volume in the TMF (100 mg/kg) + DDP (2 mg/kg) group was lower than that in the DDP (2 mg/kg) group, but it did not reach a statistical difference between them (*p* = 0.10). Moreover, the result of tumor weight in the seven groups was consistent with the result of the increased tumor volume (Figure 3C,F).

### 3.3 Effect of TMF on Transcriptome of HeLa Tumor

**FIGURE 4:**
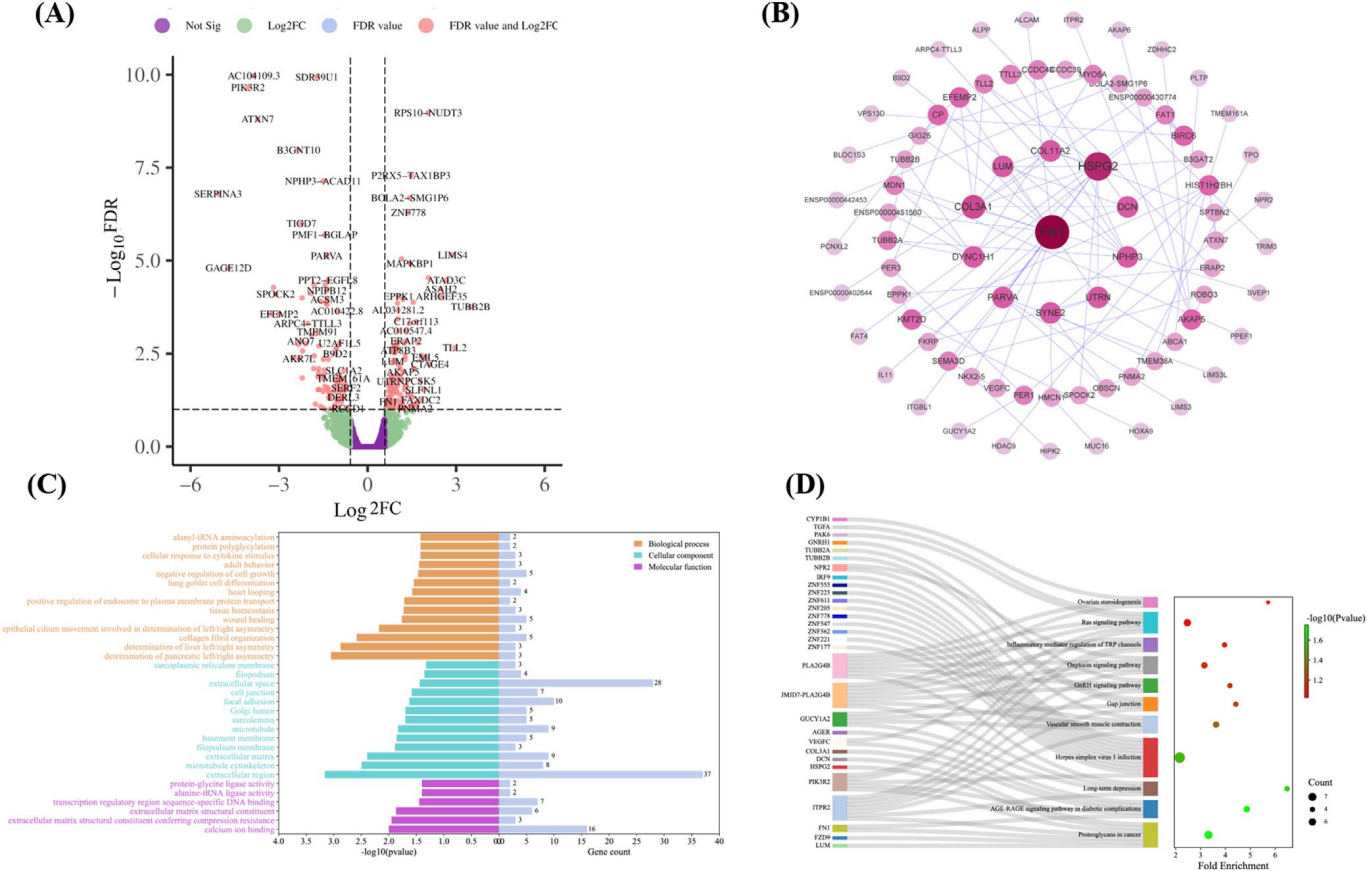
Effect of TMF (100 mg/kg) on the gene transcription levels of HeLa tumor implanted in nude mice. (A) Volcano plot representation of TMF‐induced change of gene expression levels; purple indicates no significance; green indicates FC > 1.5 and FDR > 0.1; red indicates FDR < 0.1 and FC > 1.5, Log2(FC) < 0 indicates downregulation, and Log2(FC) > 0 indicates upregulation. (B) Protein–protein interaction (PPI) analysis of these 261 differentially expressed genes (DEGs). (C) Graphical representation of Gene Ontology (GO) functional annotation analysis for these 261 DEGs; Y‐axis indicates specific GO functions including biological process, cellular component, and molecular function, where left X‐axis is the −log10(*p* value), and right X‐axis is the gene count of a corresponding GO function. (D) Cnetplot representation of KEGG pathway enrichment for these 261 DEGs.

After intraperitoneal administration of 100 mg/kg TMF into nude mice bearing subcutaneous HeLa xenografts, the transcriptional level of 261 genes was significantly changed, including 120 downregulated genes and 141 upregulated genes (Figure 4A). Based on the 261 DEGs, PPI, GO and KEGG analysis were performed. The GO classification results suggested that the top five changes induced by TMF were biological regulation (6.67%), developmental process (6.20%), growth (4.47%), single‐organism process (4.31%), and multicellular organismal process (3.81%) in biological processes; macromolecular complex (5.74%), cell junction (3.66%), synapse (3.38%), membrane part (3.18%), and cell (3.17%) in cellular component; and catalytic activity (7.04%), signal transducer activity (6.24%), molecular transducer activity (5.77%), nucleic acid binding transcription factor activity (2.83%), and transporter activity (1.37%) in molecular function (Figure 4B). The KEGG classification results revealed that 194 signaling pathways were enriched according to the 261 DEGs, and cancer‐related signaling pathways primarily included pathways in cancer, proteoglycans in cancer, VEGF, MAPK, Ras, GnRH, mTOR, Wnt, FoxO, PI3K‐Akt, and MicroRNAs in cancer, indicating that the inhibitory efficacy of TMF on HeLa tumors was involved in multipathing in vivo (Figure 4C,D).

### 3.4 Effect of TMF on Proteome of HeLa Cells

**FIGURE 5:**
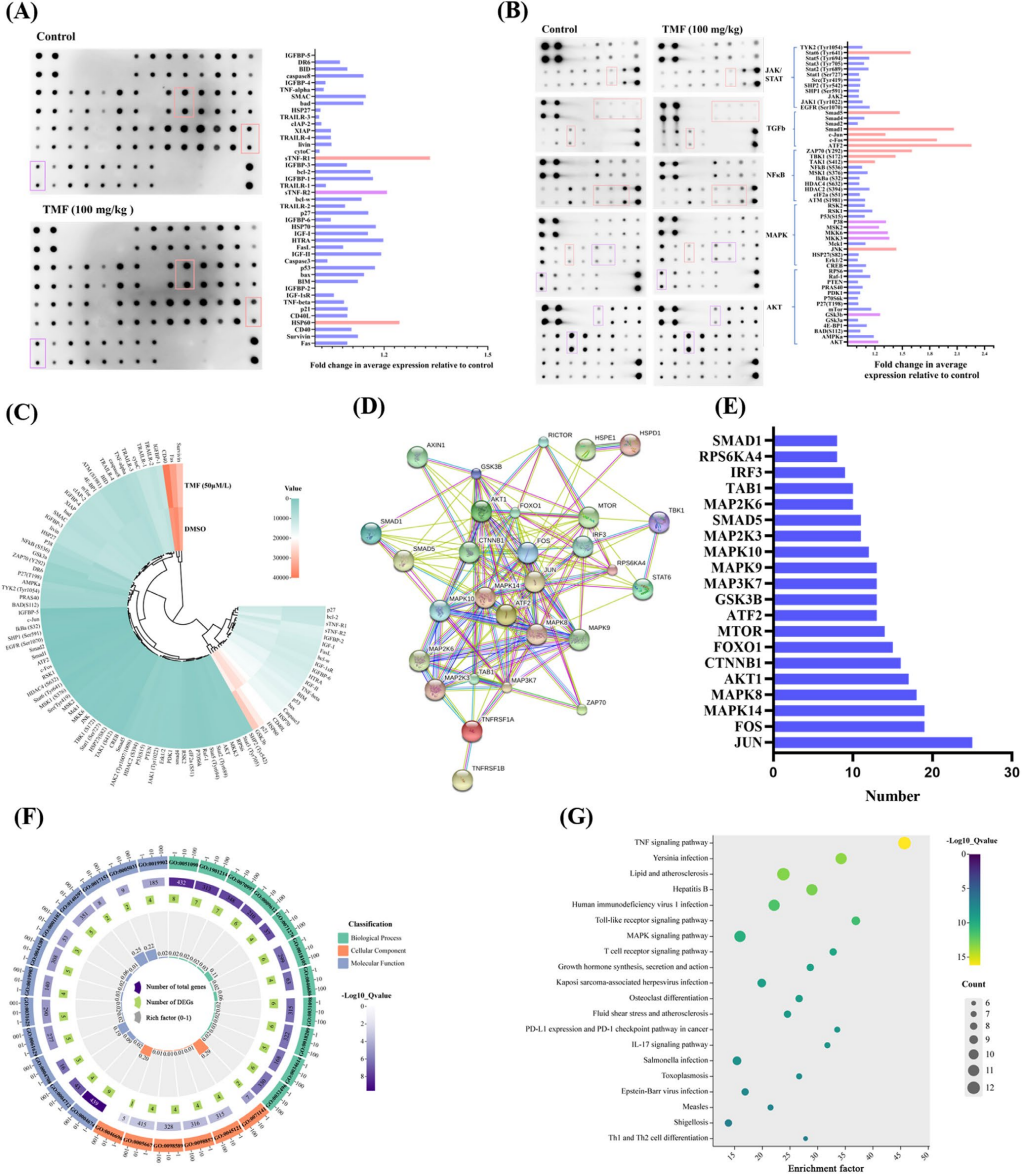
Effect of TMF on the protein expression of HeLa tumor implanted in nude mice. (A) Antibody array analysis of 43 apoptotic‐related proteins and graphic representation of their expression levels; (B) Antibody array analysis of 55 phosphorylated proteins involving AKT, MAPK, NFκB, TGFb, and JAK/STAT signaling pathways and graphic representation of their expression levels. Blue indicates no change, orange indicates upregulation, and purple indicates downregulation; (C) Doughnut heatmap representation of the expression levels of these 88 proteins; (D) Protein–protein interaction network of the 19 DEPs constructed using the STRING database; (E) Analysis of the top 10 proteins for the number of connecting nodes based on protein–protein interaction network; (F) GO function annotation of the 19 differentially expressed proteins; (G) KEGG pathway enrichment analysis based on the 19 differentially expressed proteins (DEPs).

To clarify the mechanism of action of TMF on HeLa tumors from the aspect of protein expression levels, a total of 98 proteins were determined using an antibody array, including 43 apoptosis‐associated proteins and 55 phosphorylated proteins involving AKT, MAPK, NFκB, TGFb, and JAK/STAT signaling pathways. The results suggested that the expression levels of 19 of the 98 proteins were markedly changed in TMF (100 mg/kg)‐treated mice compared to those in vehicle‐treated mice, including downregulation of HSP60, sTNF‐R1, JNK, TAK1 (S412), TBK1 (S172), ZAP70 (Y292), ATF2, c‐Fos, c‐JUN, Smad1, Smad5 and Stat6 (Tyr64), and upregulation of sTNF‐R2, AKT, GSK3b, MKK3, MKK6, MSK2, P38 (Figure 5A–C).

Based on these 19 DERs, GO function annotation results revealed that biological process was enriched primarily in apoptotic mitochondrial changes, regulation of DNA‐binding transcription factor activity, and neuron death; the cellular component was enriched primarily in transcription regulator complex, membrane region, and membrane microdomain; and molecular function was enriched primarily in protein serine/threonine kinase activity, DEAD/H‐box PNA helicase binding, and DAN‐binding transcription factor binding (Figure 5F). KEGG pathway enrichment analysis suggested that 97 signaling pathways were identified, including MAPK, TNF, and PD‐L1 expression and PD‐1 checkpoint pathway in cancer, with the MAPK signaling pathway showing the largest number of enriched DEPs, the highest significance, and the highest degree of enrichment (Figure 5G). According to the network analysis, JUN (25), FOS (19), MAPK14 (19), MAPK8 (18), AKT1 (17) were the top 5 most connected proteins (Figure 5D,E).

### 3.5 Effect of TMF on Histopathology of Mice Bearing HeLa Xenograft

**FIGURE 6:**
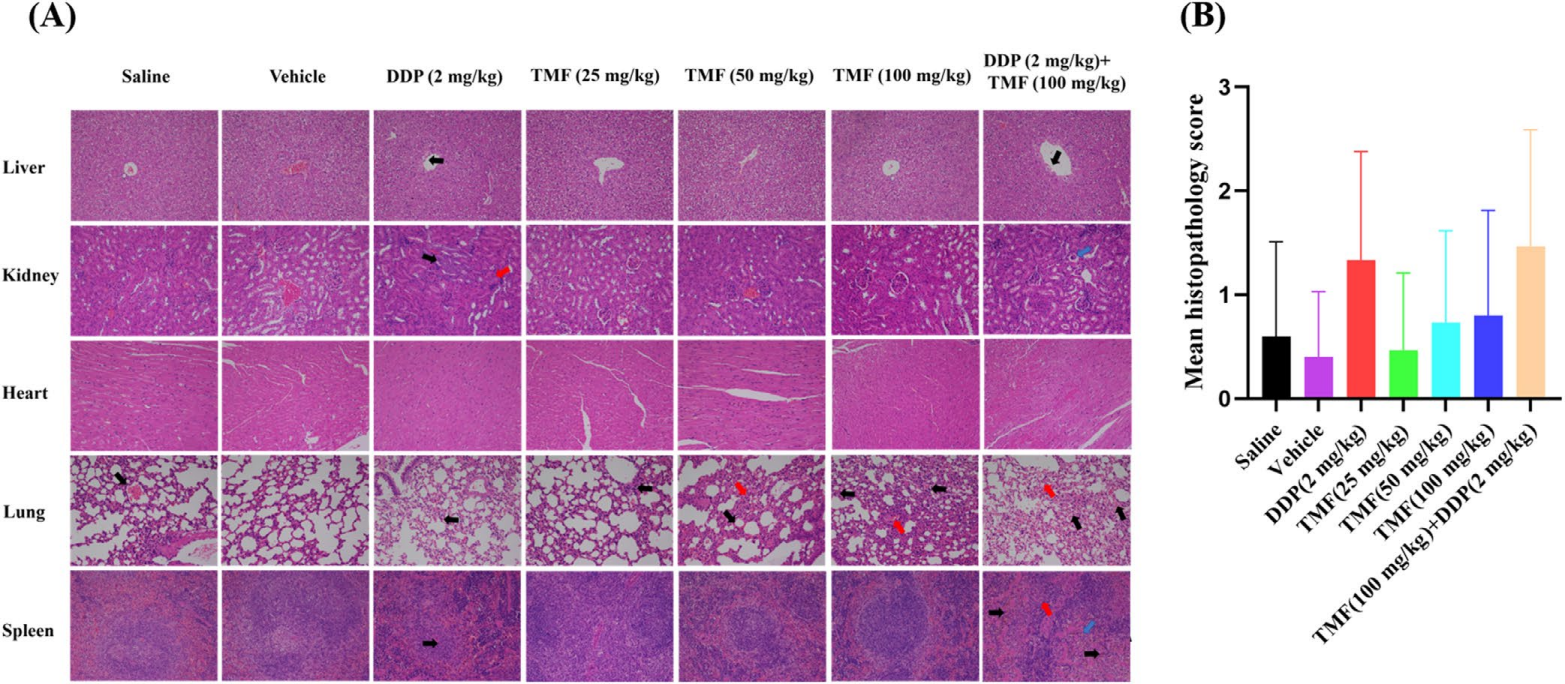
(A) Histological characterization of liver, kidney, heart, lung, and spleen of nude mice (200 times magnification). From left to right were saline, vehicle (control), DDP (2 mg/kg, positive control), TMF (25 mg/kg, low dose), TMF (50 mg/kg, medium dose), TMF (100 mg/kg, high dose), and TMF (50 mg/kg) + DDP (25 mg/kg) groups. (B) Comprehensive evaluation on the histomorphology of nude mice treated with TMF.

At the end of the experiment, the liver, kidney, heart, lung, and spleen of nude mice bearing HeLa xenografts were collected and fixed in 4% paraformaldehyde solution, and histopathological examinations of these tissues were performed to evaluate the safety of TMF on nude mice. H&E staining showed that the liver, kidney, and spleen segments of the saline, vehicle, and TMF (25, 50, and 100 mg/kg) groups preserved normal histological appearance, while those of the DDP (2 mg/kg) and DDP (2 mg/kg) + TMF (100 mg/kg) groups presented obvious histological changes, such as hepatic cell swelling, degeneration, apoptosis, necrosis, or inflammatory cell infiltration (arrow). In the heart segment, the myocardial fiber structure was normal in all groups, and no histopathological alteration was observed in any group. For lung histopathology, although histopathological alterations occurred in all but the vehicle control group, the histopathological alterations of lung hyperemia and swelling, damaged alveoli, alveolar rupture, necrosis, and bleeding were much more pronounced in the DDP (2 mg/kg) and DDP (2 mg/kg) + TMF (100 mg/kg) groups. Meanwhile, the comprehensive evaluation results suggested that the mean histopathological scores of the seven groups from left to right were 0.60, 0.40, 1.33, 0.47, 0.73, 0.80, and 1.47, respectively, and DDP (2 mg/kg) and DDP (2 mg/kg) + TMF (100 mg/kg) were much higher than other groups, indicating TMF was much less toxic than DDP, as shown in Figure 6.

### 3.6 Effect of TMF on Plasma Biochemical Indices of Mice Bearing HeLa Xenograft

To further assess the safety of TMF in vivo, biochemical indices related to liver function (ALT, AST, TBIL, DBIL, TRIG, ALP, LDH, and GGT), kidney function (CREA, UA, and UREA), and heart function (CK, HBD, and CHOL) were selected and determined in this study.

**FIGURE 7:**
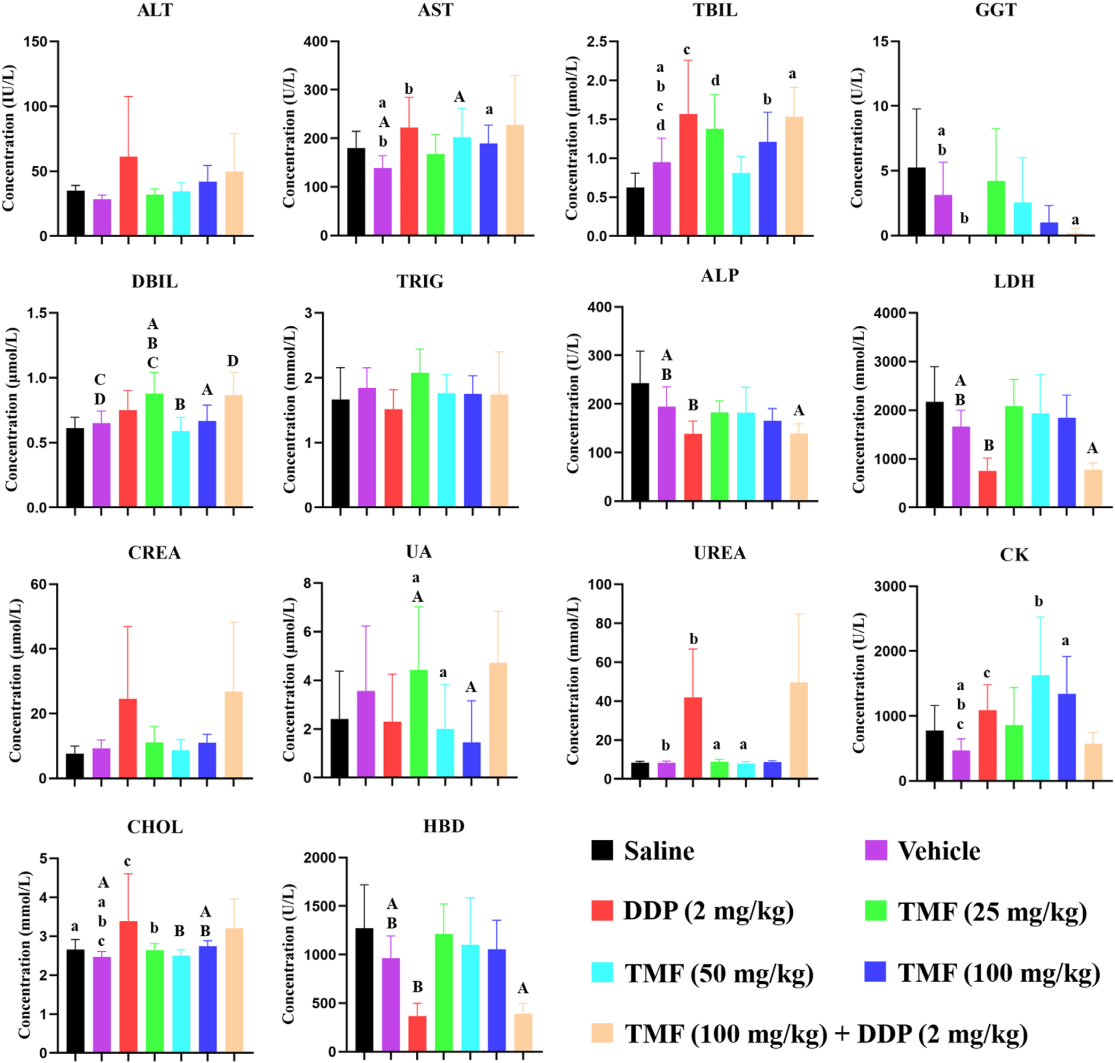
Plasma biochemical index levels including ALT, AST, TBIL, DBIL, TRIG, ALP, LDH, GGT, CREA, UA, UREA, CK, HBD, and CHOL. The same superscript letter indicates that the comparison between two groups is statistically significant, where the same superscript lowercase letter indicates *p* < 0.05 and the same superscript capital letters indicate *p* < 0.01.

As shown in Figure 7 and Table 1, the mean ALT levels in the DDP (2 mg/kg) group and DDP (2 mg/kg) + TMF (100 mg/kg) group were visibly higher than in the other groups. The plasma AST levels increased with the increase of TMF concentration among the control group, TMF (25 mg/kg) group, TMF (50 mg/kg) group, and TMF (100 mg/kg) group, while there was no significant difference between the control group and TMF (25 mg/kg) group (*p* > 0.05). Compared with the control group, the AST levels of the TMF (100 mg/kg) group (*p* < 0.05), TMF (50 mg/kg) group (*p* < 0.01), and DDP (2 mg/kg) group (*p* < 0.05) were significantly increased. Except for the TMF (50 mg/kg) group, TBIL in other TMF groups was significantly higher than in the control group (*p* < 0.05), and the variation trend of DBIL levels was consistent with that of TBIL. There was no significant difference among all groups in terms of plasma TRIG (*p* > 0.05). Compared with the control group, both the DDP (2 mg/kg) group (*p* < 0.01) and DDP (2 mg/kg) + TMF (100 mg/kg) group (*p* < 0.01) showed significantly decreased plasma ALP and LDH, whereas the TMF (25 mg/kg), TMF (50 mg/kg), and TMF (100 mg/kg) groups showed no significant changes (*p* > 0.05). For the GGT levels, it was undetectable in the DDP (2 mg/kg) group and DDP (2 mg/kg) + TMF (100 mg/kg) group. The above results demonstrated that DDP induced obvious liver dysfunction, which was reflected by increased plasma ALT, AST, and TBIL, and decreased plasma GGT, ALP, and LDH, while TMF showed a limited effect on liver function.

**TABLE 1:** Plasma biochemical indexes related to liver, kidney, and myocardium function.

Biochemical indexes	Saline (*n* = 8)	Vehicle (*n* = 8)	DDP (*n* = 6) (2 mg/kg)	TMF (*n* = 9) (25 mg/kg)	TMF (*n* = 9) (50 mg/kg)	TMF (*n* = 9) (100 mg/kg)	TMF (100 mg/kg) + DDP (2 mg/kg) (*n* = 6)
ALT (U/L)	35.1 ± 3.8	28.5 ± 3.0	61.4 ± 42.3	32.1 ± 4.1	34.6 ± 6.2	42.0 ± 11.7	49.8 ± 26.8
AST (U/L)	180.4 ± 31.9	138.8 ± 24.1^a,A,b^	222.1 ± 57.1^b^	168.2 ± 37.3	202.6 ± 56.3^A^	189.9 ± 35.2^a^	228.0 ± 93.1
TBIL (μmol/L)	0.6 ± 0.2	1.0 ± 0.3^a,b,c,d^	1.6 ± 0.6^c^	1.4 ± 0.4^d^	0.8 ± 0.2	1.2 ± 0.4^b^	1.5 ± 0.3^a^
DBIL (μmol/L)	0.6 ± 0.1	0.7 ± 0.1^C,D^	0.8 ± 0.1	0.9 ± 0.2^A,B,C^	0.6 ± 0.1^B^	0.7 ± 0.1^A^	0.9 ± 0.2^D^
TRIG (mmol/L)	1.7 ± 0.5	1.8 ± 0.3	1.5 ± 0.3	2.1 ± 0.3	1.8 ± 0.3	1.8 ± 0.3	1.7 ± 0.6
ALP (U/L)	243.3 ± 61.2	194.5 ± 38.4^A,B^	138.7 ± 23.9^B^	182.4 ± 22.5	182.1 ± 49.5	165.6 ± 23.5	139.0 ± 18.8^A^
LDH (mmol/L)	2172.5 ± 675.8	1665.4 ± 314.3^A,B^	754.3 ± 242.1^B^	2081.1 ± 518.7	1935.0 ± 755.5	1843.8 ± 441.7	779.0 ± 125.1^A^
GGT (U/L)	5.3 ± 4.2	3.1 ± 2.4^a,b^	0^b^	4.2 ± 3.8	2.6 ± 3.3	1.0 ± 1.2	0.2 ± 0.4^a^
CREA (μmol/L)	7.6 ± 2.2	9.3 ± 2.4	24.6 ± 20.4	11.1 ± 4.7	8.7 ± 3.1	11.0 ± 2.5	26.8 ± 19.5
UA (μmol/L)	2.4 ± 1.8	3.6 ± 2.5	2.3 ± 1.8	4.4 ± 2.5^A,a^	2.0 ± 1.7^a^	1.5 ± 1.6^A^	4.7 ± 1.9
UREA (mmol/L)	8.3 ± 0.8	8.3 ± 0.9^b^	42.0 ± 22.7^b^	8.9 ± 1.2^a^	8.0 ± 0.8^a^	8.7 ± 0.6	49.6 ± 32.1
CK (U/L)	772.6 ± 365.5	467.8 ± 169.3^a,b,c^	1090.7 ± 359.2^c^	862.0 ± 546.5	1629.3 ± 845.1^b^	1344.0 ± 542.1^a^	573.6 ± 157.0
HBD (U/L)	1271.9 ± 418.4	963.5 ± 213.9^A,B^	368.3 ± 118.7^B^	1213.0 ± 292.2	1102.8 ± 456.2	1056.0 ± 281.1	391.9 ± 97.8^A^
CHOL (mmol/L)	2.7 ± 0.2^a^	2.5 ± 0.1^A,a,b,c^	3.4 ± 1.1^c^	2.6 ± 0.2^b^	2.5 ± 0.1^B^	2.7 ± 0.1^A,B^	3.2 ± 0.7

*Note:* The same superscript letter indicates that the comparison between two groups is statistically significant, where the same superscript lowercase letter indicates *p* < 0.05 and the same superscript capital letters indicate *p* < 0.01.

The DDP (2 mg/kg) group and DDP (2 mg/kg) + TMF (100 mg/kg) group showed notably increased CREA, UA, and UREA levels compared to other groups. Compared with the control group, there was no significant difference in the TMF (25 mg/kg) group, TMF (50 mg/kg) group, and TMF (100 mg/kg) group, suggesting that TMF had no effect on renal function in nude mice.

The plasma CK levels in the TMF (25 mg/kg) group, TMF (50 mg/kg) group, and TMF (100 mg/kg) group were significantly higher than those of the control group (*p* < 0.05). For HBD, the DDP (2 mg/kg) group and DDP (2 mg/kg) + TMF (100 mg/kg) group were decreased significantly compared to the control group (*p* < 0.01), while the mean CHOL levels in the DDP (2 mg/kg) group and DDP (2 mg/kg) + TMF (100 mg/kg) group were markedly increased.

## 4 Discussion

PMFs were a unique group of flavonoids with at least four methoxy groups (CEN 1991). Compared with familiar flavonoids such as quercetin, luteolin, and kaempferol, PMFs have greatly improved intestinal absorption, metabolic stability, and bioavailability due to their metabolic resistance to P450 metabolic enzymes (Wen and Walle 2006). Numerous studies have reported that PMFs show a potent inhibitory effect on various tumors, such as lung cancer, colorectal cancer, breast cancer, gastric cancer, prostate cancer, ovarian cancer, and glioma (Chen and Dong 2017). Although more than 250 natural PMFs have been identified, only a few PMFs with high content have been extensively investigated. For example, nobiletin, the most familiar PMF, has been reported to exhibit a therapeutic effect in vivo for the treatment of lung cancer (Da et al. 2016; Feng et al. 2020), colon cancer (Suzuki et al. 2004; Wu et al. 2015), prostate cancer (Tang et al. 2011, 2007), ovarian cancer (Chen et al. 2015), nasopharynx cancer (Chien et al. 2015), glioma (Zhang et al. 2017), and renal carcinoma (Wei et al. 2019). However, some less reported PMFs are more effective than nobiletin and tangeretin in certain tumor models. TMF, a member of the PMF family, is present dominantly in Citrus species (Xu et al. 2019). Many studies have demonstrated its anticancer activity. Our previous study found that TMF was more potent in inhibiting HeLa cell proliferation than nobiletin and tangeretin in vitro. However, it remains unclear whether TMF exerts anti‐HeLa cancer effects in vivo. In this work, a subcutaneous xenograft model in nude mice was established to investigate the therapeutic efficacy of TMF against HeLa cancer in vivo.

In the present study, we first determined the apoptosis‐inducing effect of TMF using flow cytometry. The results indicated that TMF significantly increased the proportion of early and late apoptotic HeLa cells in a dose‐dependent manner compared to the control. Prior to this work, other PMFs, including zapotin (5,6,2′,6′‐tetramethoxyflavone) (Toton et al. 2016) and casticin (5,3′‐dihydroxy‐3,6,7,4′‐tetramethoxyflavone), have been reported to induce apoptosis in HeLa cells via reactive oxygen species‐dependent sustained activation of Jun N‐terminal kinase (Chen et al. 2011; Zeng et al. 2012). Then, the xenograft model was used to evaluate the efficacy of TMF against HeLa tumors in vivo. According to the results of the in vivo experiment, TMF significantly suppressed HeLa tumor growth implanted in nude mice in a dose‐dependent manner. Both the mean tumor volume and tumor weight of the DDP (2 mg/kg) group were slightly lower than those of the DDP (2 mg/kg) + TMF (100 mg/kg) group, but they did not differ significantly, suggesting that DDP and TMF showed no synergistic therapeutic effect against HeLa tumors. Among the seven groups, two mice died in the DDP (2 mg/kg) group and three mice died in the DDP (2 mg/kg) + TMF (100 mg/kg) group during the experiment. The body weight in the two groups was significantly lower than in the other five groups, indicating that DDP caused serious toxicity to mice. Clinically, the severe side effects of cisplatin have been a main obstacle to satisfactory therapeutic efficacy (Yan et al. 2021), whereas the behavior, diet and body weight of mice did not change significantly in the low‐, medium‐, and high‐dose TMF groups, compared to the control group.

After evaluating the antitumor activity of TMF in vivo, proteomics and transcriptomics were used to reveal the mechanism of action of TMF against HeLa tumors. Of the 98 proteins determined, 12 proteins, including HSP60, sTNF‐R1, JNK, TAK1 (S412), TBK1 (S172), ZAP70 (Y292), ATF2, c‐Fos, c‐JUN, Smad1, Smad5, and Stat6 (Tyr64), were significantly downregulated, and 7, including sTNF‐R2, AKT, GSK3b, MKK3, MKK6, MSK2, and P38, were significantly upregulated. KEGG enrichment analysis revealed that the therapeutic effect of TMF on the HeLa tumor was involved in multi‐signaling pathways such as \MAPK, TNF, and PD‐L1 expression and the PD‐1 checkpoint pathway in cancer. PMFs‐induced tumor cell inhibition via the MAPK/P38 signaling axis has been widely investigated in previous studies (Abe and Yuasa 2019; Lien et al. 2016; Zhong et al. 2014). Meanwhile, the transcriptomics study further evidenced that 261 DEGs were primarily enriched in several signaling pathways including VEGF, MAPK, Ras, and pathways in cancer, which were consistent with the proteomics results.

TMF was also a dietary component presented in orange juice (Glabasnia et al. 2018; Leuzzi et al. 2000). In this study, TMF was administered intraperitoneally to nude mice for 15 days, and its maximum dose given was 100 mg/kg. Therefore, evaluating its safety is of necessity. According to histopathology results, high‐dose TMF (100 mg/kg) did not result in histopathology alterations of the liver, kidney, heart, lung, and spleen of nude mice, while DDP (2 mg/kg) significantly altered the histopathology of the liver, kidney, lung and spleen of nude mice. In addition, analysis of biochemical indexes verified that most of the 14 biochemical indexes were abnormal in DDP‐administered groups, which were highly consistent with the histopathology results. Overall, TMF (100 mg/kg) alone did not affect the body weight, mortality, histopathology and main clinical biochemical indexes in nude mice, indicating that TMF was a safe compound for the treatment of HeLa cancer.

## 5 Conclusion

This study demonstrates that TMF exerts potent antitumor effects against HeLa xenografts by modulating MAPK, TNF, VEGF, Ras, and FoxO pathways via multi‐target regulation, as evidenced by proteomic and transcriptomic analyses. Crucially, TMF administration induced no histopathological damage in vital organs and elicited significantly milder systemic biomarker alterations compared to cisplatin, suggesting its superior safety profile. These findings collectively characterize TMF as a candidate demonstrating an optimal efficacy–safety balance for cervical cancer treatment, warranting further preclinical development.

## Author Contributions

**Qiang You:** formal analysis (equal), funding acquisition (equal), investigation (equal), visualization (equal), writing – original draft (equal). **Haiyan Ding:** data curation (equal), investigation (equal), methodology (equal). **Dan Li:** formal analysis (equal), visualization (equal). **Yuan Hu:** project administration (equal), writing – review and editing (equal). **Youping Liu:** conceptualization (equal), supervision (equal), writing – review and editing (equal).

## Conflicts of Interest

The authors declare no conflicts of interest.

## Data Availability

Data will be made available on request.
